# Gastric Schwannoma as an Important and Infrequent Differential Diagnosis of Gastric Mesenchymal Tumours: A Case Report and Review of Literature

**DOI:** 10.7759/cureus.32112

**Published:** 2022-12-01

**Authors:** Abdalla Saad Abdalla Al-Zawi, Salma Lahmadi, Saman Jalilzadeh Afshari, Ipshita Kak, Salem Alowami

**Affiliations:** 1 General and Breast Surgery, Mid and South Essex University Hospital Group, Basildon, GBR; 2 General and Breast Surgery, Basildon and Thurrock University Hospital, Basildon, GBR; 3 General and Breast Surgery, Anglia Ruskin University, Chelmsford, GBR; 4 Surgical Oncology, The National Institute of Oncology, Rabat, MAR; 5 Ambulatory Assessment Unit, Oxford University Hospitals NHS Foundation Trust, Oxford, GBR; 6 Pathology, McMaster University, Hamilton, CAN

**Keywords:** cd117, gfap, s-100, neurofibromatosis ii, gist, gastric schwannoma

## Abstract

The spectrum for gastrointestinal tract mesenchymal tumours includes leiomyomas, leiomyosarcomas, gastrointestinal stromal tumours (GISTs) and schwannomas. Schwannomas (also known as neuroma, neurilemmomas or neurinomas of Verocay) are well-known slow-growing, benign neoplasms that originate from nerve plexuses within a Schwann cell sheath. They can arise anywhere along the course of the peripheral nerve and are frequently reported around the head and neck, brachial plexus and along the gastrointestinal tract. Usually, these tumours are detected as solitary; however, they can occur at multiple sites around the body. Schwannomatosis (multiple schwannomas) is usually associated with neurofibromatosis type 2; the pathogenesis is triggered by mutations of the neurofibromatosis 2 tumour suppressor gene resulting in a loss of its function. Solitary gastric schwannomas are rare lesions that arise from the nerve plexus of the gastric wall. Frequently they are detected incidentally or may present with nonspecific abdominal pain or bleeding. This paper reports the case of a 79-year-old patient diagnosed with gastric schwannoma after presenting with abdominal pain.

Gastric schwannomas should be taken into consideration while making a differential diagnosis of lesions that are gastric mesenchymal tumours, which span a broad spectrum. Gastric schwannomas are typically benign, considerably less common than gastric GISTs, and have an excellent prognosis following excision.

## Introduction

Gastrointestinal tract mesenchymal tumour subtypes include leiomyoma, leiomyosarcoma, gastrointestinal stromal tumours (GISTs) and schwannomas. GISTs form the largest group of gut mesenchymal tumours [1]. Gut schwannomas are rarer, benign, homogeneous and slow-growing spindle cell mesenchymal lesions that originate from the Schwann cells of the peripheral nerve sheath. Gastric schwannomas account for 6.3% of gut mesenchymal tumours and are thought to originate from Auerbach’s plexus, or less frequently from Meissner’s plexus nerve sheaths [2]. Histologically, schwannomas are characterised by the presence of singular architectural patterns known as Antoni A (hyper-cellular area) and Antoni B (hypo-cellular areas) [3]. The pre-operative diagnosis could be a real challenge to the clinician and in many cases, surgical excision is needed to elicit the final diagnosis.

## Case presentation

**Figure 1:**
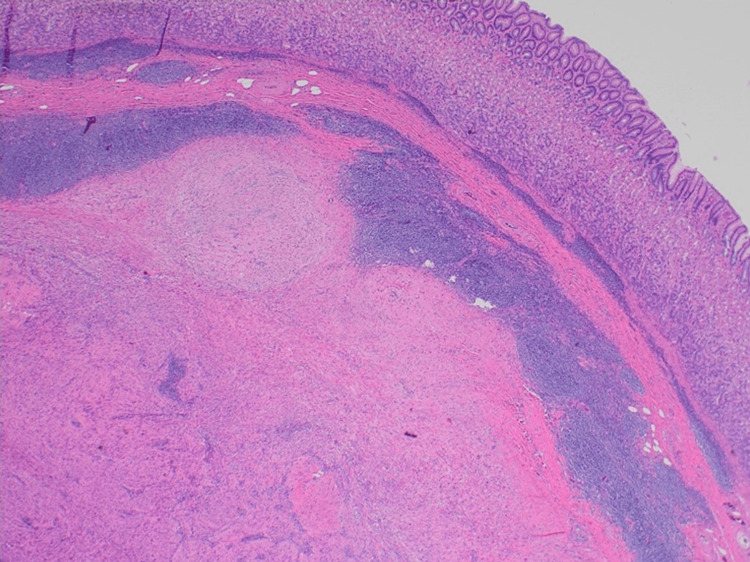
Gastric schwannoma – nodule of spindle cell tumour separated from the mucosa by a cuff of lymphoid cells, x20 haematoxylin and eosin stain (HE).

**Figure 2:**
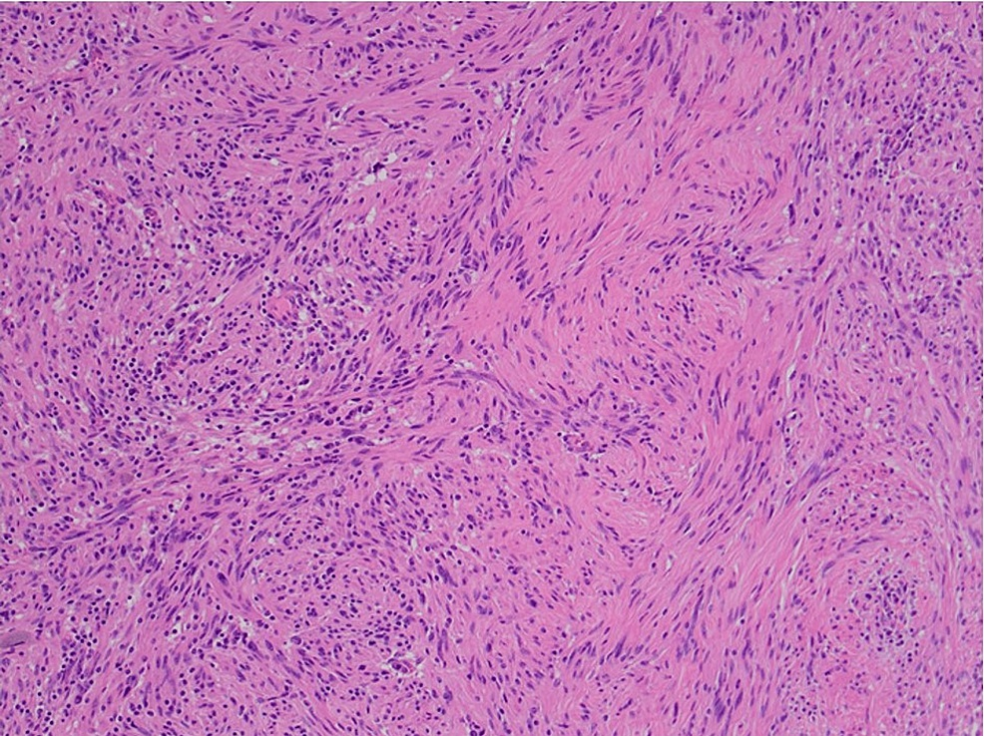
Gastric schwannoma – benign spindle cell proliferation, x100 HE.

**Figure 3:**
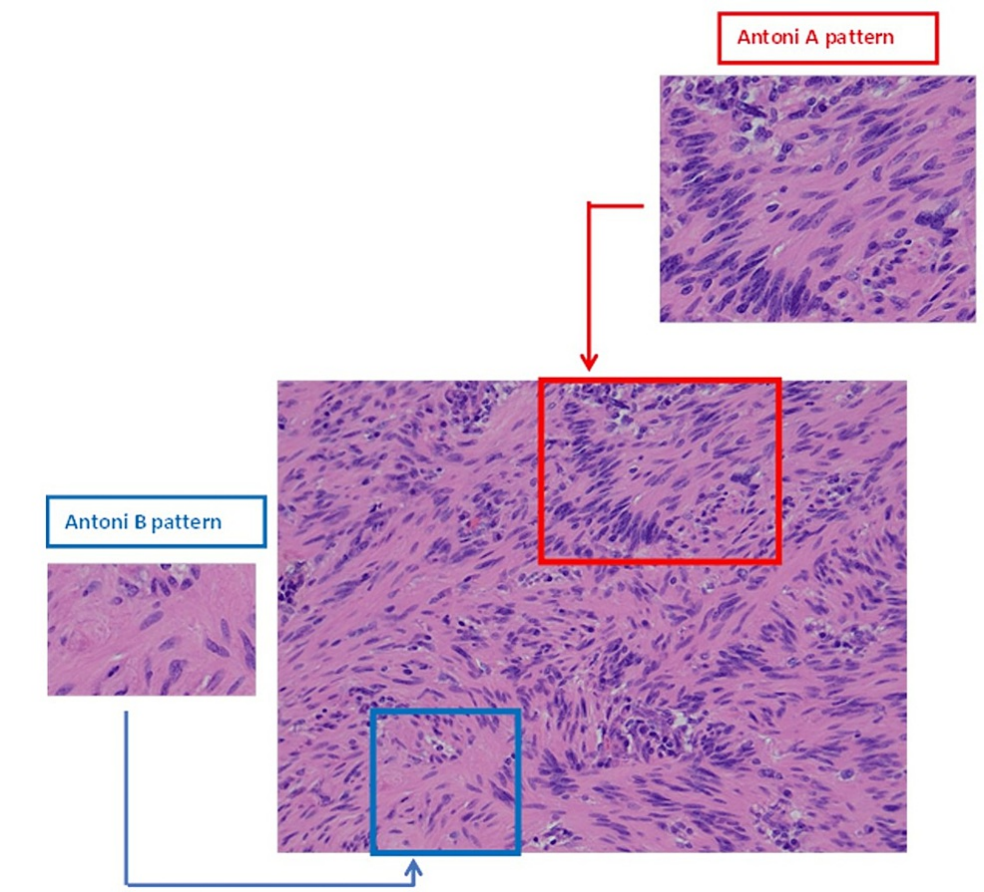
Gastric schwannoma – nuclear palisade with hypo and hyper-cellular areas (Antoni type A and Antoni type B), x200 HE.

**Figure 4:**
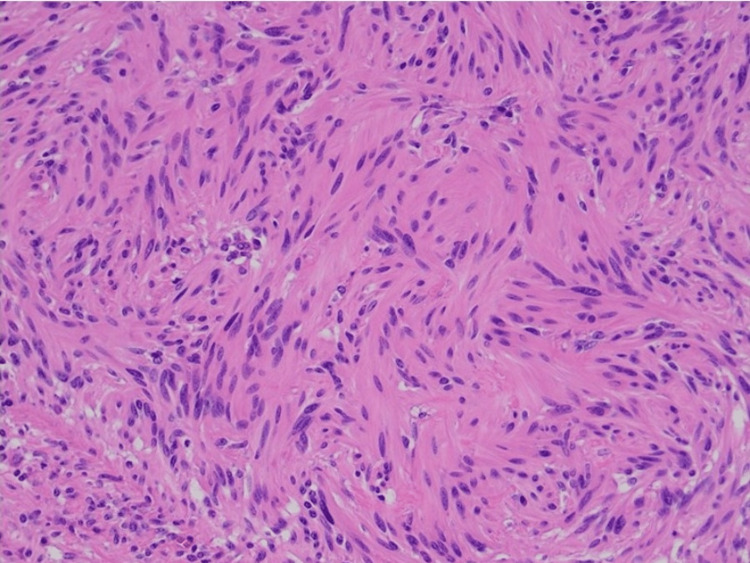
Gastric schwannoma – benign spindle cell proliferation with a lack of atypia, mitosis and necrosis, X 200 HE.

A 79-year-old female presented with abdominal pain. Relevant past medical history included hypercholesterolemia and hypertension with a previous angioplasty. The patient had no known drug allergies and was taking aspirin, amlodipine, atorvastatin, bisoprolol, valsartan and lamotrigine. On clinical examination, the abdomen was soft and non-tender in all quadrants. A digital rectal examination did not reveal any concerning lesions. A computerised tomography (CT) scan showed a 3.2 x 2.3 cm exophytic isodense mass arising from the body of the stomach, likely representing a GIST. Gastroscopy demonstrated a 3-5 cm mass located in the greater curvature of the stomach and biopsies were obtained. The remainder of the gastroscopy and colonoscopy were unremarkable. Biopsies demonstrated mild chronic gastritis with no other abnormality, with a comment to ascertain if these biopsies were representative of the mass. The patient was brought in for laparoscopic sleeve gastrectomy and resection of the submucosal mass in the greater curvature. A gross examination of the pathological specimen revealed a soft, circumscribed submucosal mass extending to the serosa and measuring 3.0 x 2.8 x 2.0 cm. Microscopic examination showed a spindle cell tumour, surrounded by a lymphoid cuff (Figures 1, 2) with neural characteristics of hypo-and hypercellular areas (Antoni A and Antoni B patterns, Figure 3). There was no cellular atypia, mitosis or necrosis noted (Figure 4).

 

 

**Figure 5:**
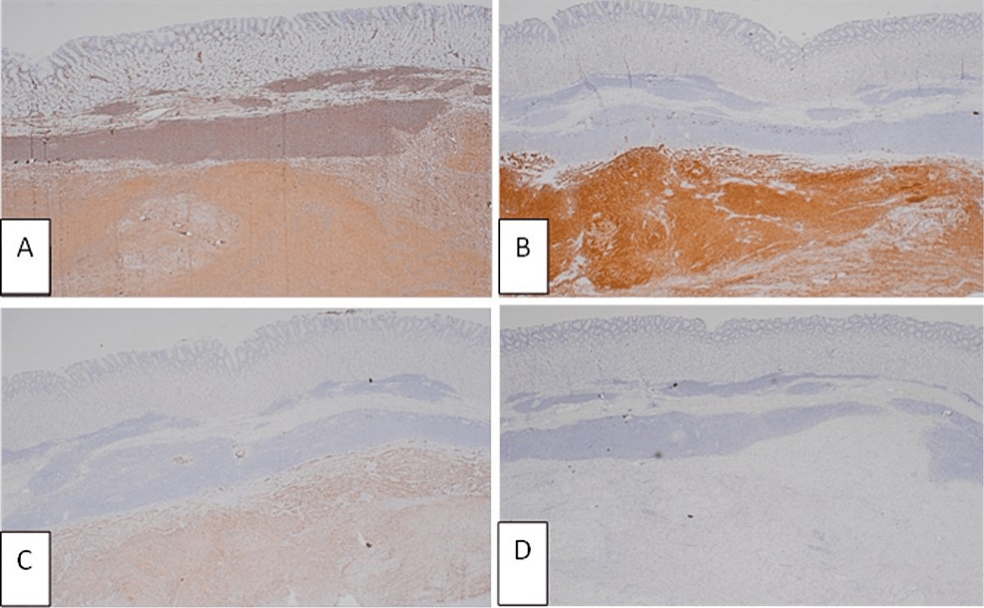
Gastric schwannoma, x20 – (A) positive staining with Vimentin; (B) positive staining with S-100; (C) positive staining with GFAP; (D) negative staining with c-KIT.

**Figure 6:**
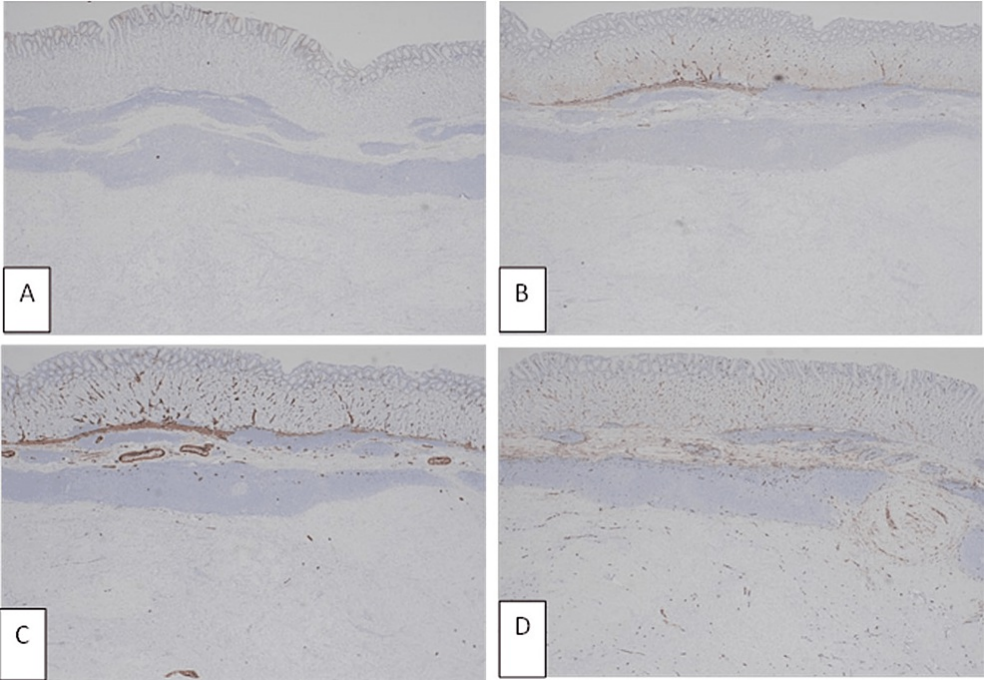
Gastric schwannoma, x20 – (A) negative staining with DOG-1; (B) negative staining with Desmin; (C) negative staining with Caldesmon; (D) negative staining with CD34.

On immunohistochemistry, the tumour cells were strongly positive for Vimentin (Figure 5A), S100 (Figure 5B) and Glial fibrillary acidic protein (GFAP) (Figure 5C). The tumour cells were negative for c-KIT (Figure 5D), DOG-1 (Figure 6A), Desmin (Figure 6B), H-caldesmon (Figure 6C) and CD34 (Figure 6D). The histology and immune-immune profile were characteristic of a gastric schwannoma. The margins of the specimen were clear, and the surrounding gastric mucosa was unremarkable. Follow-up abdominal CT scan performed was compatible with complete resection of the gastric tumour. Repeat gastroscopy was normal with evidence of prior surgery. Routine biopsies from this gastroscopy showed gastric mucosa with reactive changes and no residual tumour, the patient remains under surveillance.

## Discussion

**Figure 7:**
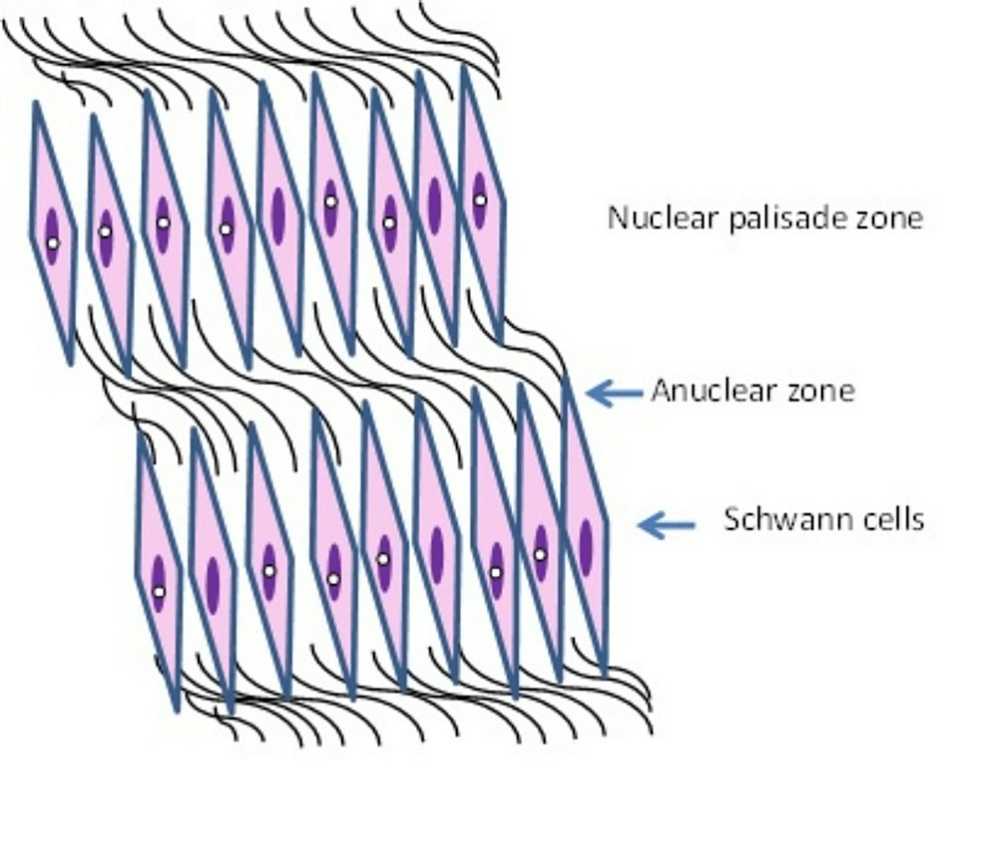
Antoni A areas are characterised by interlacing bundles of spindle-shaped Schwann cells with oval or wavy nuclei, eosinophilic cytoplasm, and indistinct cytoplasmic borders. Also intranuclear vacuoles may be present in some cells. Image credit: Abdalla Saad Abdalla Al-Zawi

**Figure 8:**
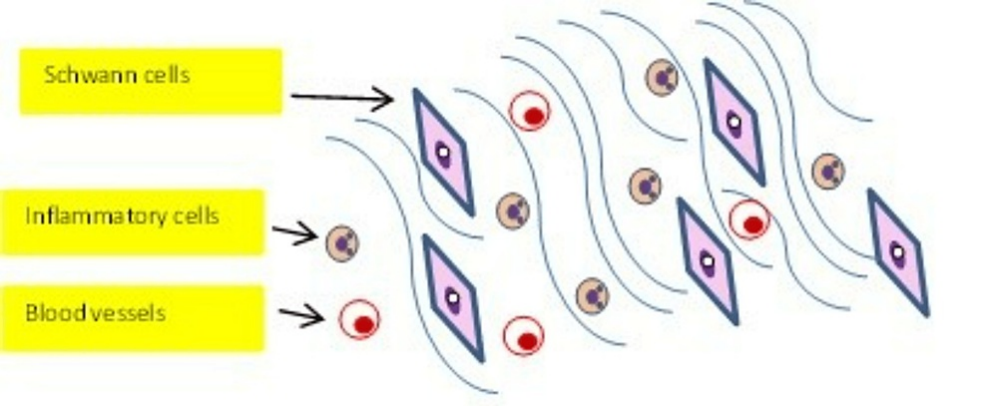
Antoni B areas are less compact than Antoni A areas and hypocellular. They are composed of haphazardly arranged spindle cells in a loose myxoid or hyalinized stroma containing a few inflammatory cells and delicate collagen fibres. Antoni B areas also contain variably sized, deformed blood vessels which frequently have hyalinized walls and contain thrombi. Image credit: Abdalla Saad Abdalla Al-Zawi

The stomach may harbour different types of lesions, and their pathology may be consistent with benign epithelial tumours, stromal lesions or malignant epithelial tumours which are related to a cancer mortality rate almost similar to breast cancer with an incidence of 7%-8% [4]. Peripheral nerve sheath tumours (PNST) are uncommon soft tissue nerve tumours with schwannomas being the most frequent lesions. Schwannomas are benign, encapsulated tumours of peripheral nerve sheath Schwann cells that produce myelin. They were first described by Uruguayan neuro-pathologist Jose Verocay in 1908 who labelled them “neurinomas” [5]. In 1968, Harkin and Reed introduced the term “schwannoma” to describe these perineural, circumscribed lesions. Although they are most frequently encountered in the cranial vault, they can also arise from the brachial plexus, upper limbs, stomach and masticator space [6]. Histologically, they consist of different types of cells (tumorigenic Schwann cells, nerve axons, T cells, macrophages and fibroblasts) in addition to vascular structures and the extracellular matrix. Morphologically, they are also characterised by the presence of singular architectural patterns called Antoni A and Antoni B areas (Figures 7, 8), this concept was first described by the Swedish neurologist Nils Antoni in 1920 [3].

Antoni A pattern is highly cellular and exhibits palisades of Schwann cell nuclei surrounded by prominent extracellular matrix and laminin secretion (also known as Verocay bodies). Laminin is a glycoprotein found in the basement protein of the Schwann cells and plays a major part in axon myelination and nerve injury regeneration. A typical Verocay body is composed of a stacked display of two chains of longitudinal palisading nuclei that alternate with acellular areas made up of the Schwann cells’ cytoplasmic processes (Figure 7). Antoni B pattern is characterised by loosely organized tissue with cystic and myxomatous changes (Figure 8) - some authors consider this to represent Antoni A pattern degeneration.

These tumours usually are detected as solitary lesions; however, they can occur at multiple sites of the body. Schwannomatosis (multiple schwannomas) is usually associated with neurofibromatosis type 2 [7]. Neurofibromatosis type 2 (NF2) tumour suppressor gene deficiency in Schwann cells, believed to be the tumorigenic factor in neoplasm development, is increasingly targeted in research in relation to the pathogenesis of schwannoma. The most frequent site of gut schwannomas is the stomach (60%) where it represents 0.2% of all gastric tumours [8,9]. The second most common site is the colon. Gut schwannomas have been reported in the small intestine, oesophagus and rectum. They are commonly encountered between the fourth and sixth decade of life with a slight female predominance. Usually solitary lesions, they originate in the gastric lesser curvature but also frequently at the middle third of the stomach and are relatively small in size (<5 cm). Normally, they are asymptomatic and detected incidentally. As they are slow growing, they displace nerve branches away from the tumour, and thus, the neural function in the neighbouring structures is maintained. Symptomatic gastric schwannomas can present with a variety of symptoms that can be attributed to the tumour’s enlargement and the compression or injury of neighbouring structures.

They can present as mass lesions, but the most frequent symptoms are upper gastrointestinal bleeding, abdominal pain, non-specific abdominal discomfort, nausea and gastric outlet obstruction. Ischaemic changes of the mucosa at the tumour site and acid activity may result in mucosal ulceration, leading to bleeding. Endoscopically, the vast majority of tumours are covered by intact overlaying mucosa and chiefly affect the submucosa and muscularis propria, sparing neighbouring structures. Central ulceration is detected in a significant number of tumours. Imaging with CT scan, MRI or ultrasonography may reveal homogenous, strongly contrast-enhanced lesions but no signs of tissue necrosis, intra-lesional haemorrhage, cystic spaces or soft tissue calcification, in contrast to GISTs [9]. Fine needle aspiration cytology using an endoscopic ultrasound technique may help in pre-operative diagnosis. FDG-PET (18F-fluorodeoxyglucose positron emission tomography) shows avid uptake in benign schwannomas but cannot differentiate them from GISTs or malignant lesions. Despite being benign, schwannomas have malignant transformation potential and malignant gastric schwannomas have been reported in the literature - this potential may possibly be related to the size of the tumour directly [8]. The patho-morphological features of gastric schwannomas are very indistinguishable from other soft tissue schwannomas; specifically spindle cell proliferation, nuclear palisading and variably myxoid stroma. However, gastric schwannomas are characterised by a rim of lymphoid tissue abutting the tumours [10]. The immunohistochemistry panel includes the S-100 protein which is an intracellular Ca2+ binding protein. It is strongly expressed in benign nerve sheath tumors whereas it is typically weak or negative in malignant PNST [11]. Vimentin is an intermediate filament for mesenchymal tissue expressed in schwannomas [12], and GFAP (glial fibrillary acidic protein) is an intermediate filament of astrocytes and shows strong expression in the cells of gut schwannomas [13]. All of these proteins were positive in our case (Figures 5A-5C).

CD117 (also known as c-KIT) is the gene encoding the receptor tyrosine kinase protein and is activated in GIST tumours but never expressed in gastric schwannomas [14]. DOG-1 (Discovered on GIST 1) is a chloride channel protein, a marker specific and sensitive for GIST tumours and uncommonly seen in other soft tissue tumours [14]. Desmin is a 53 kDa intermediate filament protein encoded by the DES gene and is a specific and sensitive marker for muscle cell differentiation. It is used to confirm the myogenic origin of neoplastic leiomyosarcoma lesions, it is negative in schwannomas [15]. H-caldesmon, a smooth muscle calcium binding protein, attaches to actin, tropomyosin and calmodulin proteins and plays a major role in the regulation of smooth muscle contraction. It is expressed in GISTs, but negative in schwannomas [16]. As expected, all the above markers were negative in our case (Figures 5D, 6A-6C). CD34 (human hematopoietic progenitor cell antigen) is a cell surface glycoprotein and works as an important element of intercellular adhesion. It is found in more than 90% of GISTs [13], and although negative in our case (Figure 6D), it can be detected in 40% of schwannomas [17].

The recommended treatment for schwannomas is complete excision of the tumour with clear resection margins. Surgical options include partial gastrectomy, wide local excision or wedge resection, done either in an open procedure or laparoscopically [18]. As schwannomas are mainly benign, they have an excellent prognosis with a disease-free survival (DFS) period of more than 36 months [3]. In contrast, GISTs have a low malignant potential [14] but a higher risk of recurrence after resection. Among all GISTs, gastric GISTs have the best prognosis, the overall three-year and five-year DFS after surgical resection of GISTs was 83% and 74%, respectively [19], where gastric schwannoma long-term outcome is excellent (Table 1) [10].

**Table 1:** Differential diagnosis and IHC of gastric schwannoma [2,3,8,10,13,14,15,20] GISTs: Gastrointestinal Stromal Tumours, GANTs: Gastrointestinal Autonomic Nerve Tumours

	S-100	Vimentin	CD117	CD34	DOG-1	SMA	GFAP
Schwannoma	+VE	+VE	-VE	-VE		-VE	+VE
GISTs.	+VE	+VE	+VE	+VE	+VE	+VE	-VE
GANTs.	-VE	+VE	+VE	+VE			-VE
Leiomyomas	-VE	+VE	+VE	-VE		+VE	
Neurofibroma	+VE	+VE		+VE			

## Conclusions

The gastric mesenchymal tumours include a wide spectrum and gastric schwannomas should be considered in the differential diagnosis of those lesions. The gastric schwannomas symptoms are non-specific abdominal pain as seen in our case; however, other symptoms such as fullness, nausea, vomiting, and change in bowel habits also had been reported. The immunohistochemistry panel is crucial for gastric schwannomas diagnosis as they are mostly benign, and the final diagnosis has been concluded only after tumour resection. Generally, they are much less frequent than gastric GISTs and have a good prognosis after resection.
